# Supplementary material for: The thioredoxin system determines CHK1 inhibitor sensitivity via redox-mediated regulation of ribonucleotide reductase activity
Source: Nat Commun. 2024 May 31;15:4667. doi: 10.1038/s41467-024-48076-9 (PMC11143221; doi:10.1038/s41467-024-48076-9)

The thioredoxin system determines CHK1 inhibitor sensitivity via redox-mediated regulation of ribonucleotide reductase activity

Chandra Bhushan Prasad[1], Adrian Oo[2], Yujie Liu[1], Zhaojun Qiu[1], Yaogang Zhong[1,3], Na Li[1], Deepika Singh[1], Xiwen Xin[4], Young-Jae Cho[2], Zaibo Li[5], Xiaoli Zhang[6], Chunhong Yan[7], Qingfei Zheng[1,3], Qi-En Wang[1], Deliang Guo[1,3], Baek Kim[2], Junran Zhang[1,3,8*]

[1]Department of Radiation Oncology, James Cancer Hospital and Richard J. Solove Research Institute, The Ohio State University, Columbus, Ohio-43210, USA; [2]Center for ViroScience and Cure, Department of Pediatrics, School of Medicine, Emory University, Atlanta, Georgia 30322, USA; [3]The Comprehensive Cancer Center, Center for Cancer Metabolism, The Ohio State University, Columbus, Ohio-43210, USA; [4]The Ohio State University, Ohio-43210, USA; [5]Department of Pathology, The Ohio State University Wexner Medical Center, College of Medicine, Columbus, Ohio-43210, USA; [6]Department of Biomedical Informatics, Wexner Medical Center, College of Medicine, The Ohio State University, Ohio-43210, USA; [7]Georgia Cancer Center, Augusta University, Augusta, Georgia-30912, USA; [8]The Comprehensive Cancer Center, Pelotonia Institute for Immuno-Oncology, The Ohio State University, Columbus, Ohio-43210, USA.

* Corresponding Author.

Correspondence may be addressed to Junran.zhang@osumc.edu

File contents:

1. Supplementary figures with description including gating strategies (Page 2-24)
2. Supplementary Methods (Page-25)
3. Uncropped western blot related to supplementary data (Page 26-29)

Figure S1. Trx1 and TrxR1 is highly expressed in NSCLC tumor samples and is associated with poor prognosis.

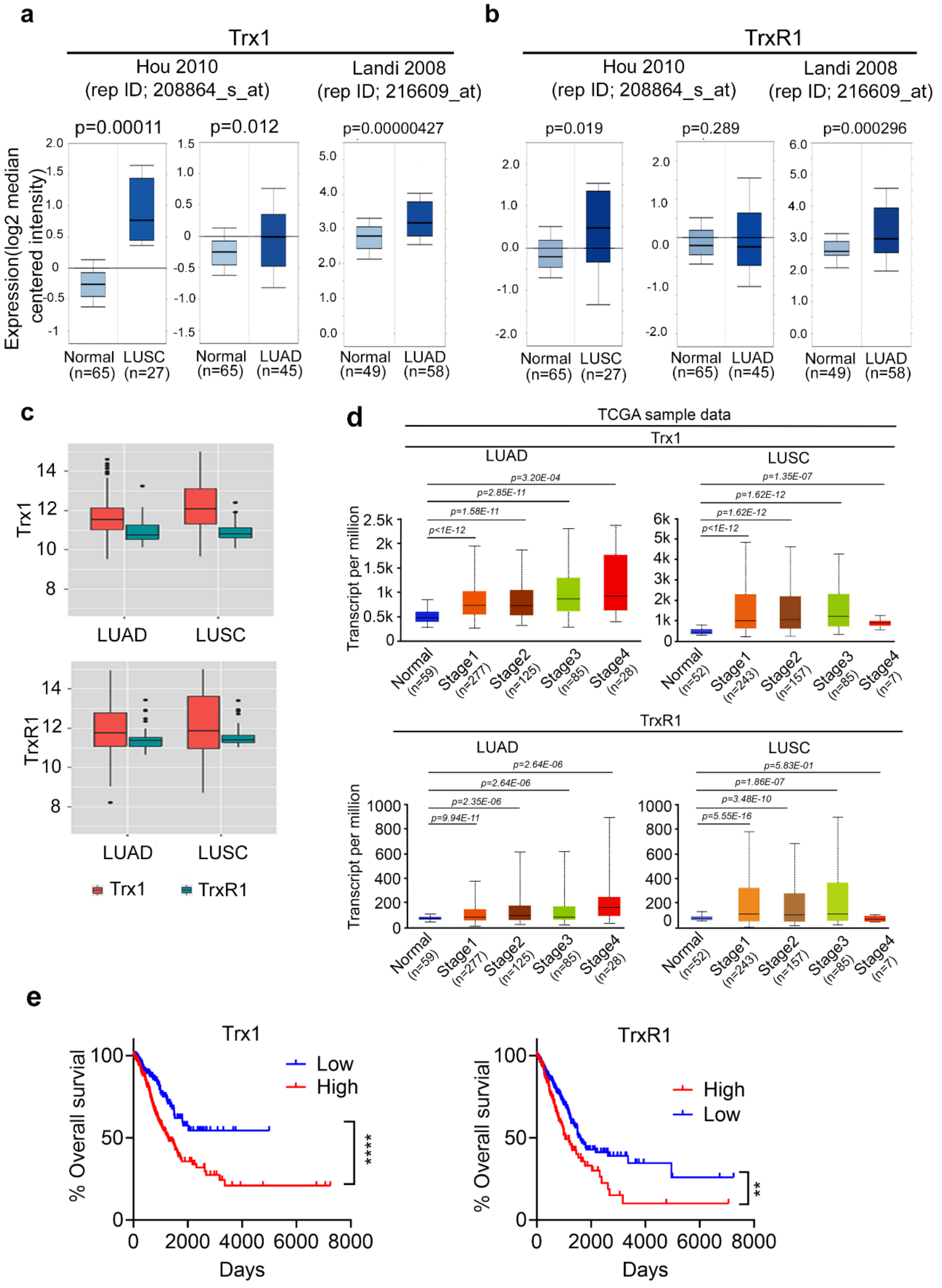

(a). Expression profile of Trx1 in NSCLC subtypes lung adenocarcinoma (LUAD) and lung squamous cell carcinoma (LUSC) that represent different datasets obtained from the Oncomine database. *P* value was determined by a two-tailed Mann–Whitney test.

(b). Expression profile of TrxR1 in LUAD and LUSC that represents different datasets obtained from the Oncomine database. *P* value was determined by a two-tailed Mann–Whitney test.

(c). Expression of Trx1 and TrxR1 in LUAD (N=560; Trx1 p<.00001; TrxR1 p<.00001) and LUSC (N=546; Trx1 <.00001; TrxR1 <.00001), compared to normal tissue. The expression of both Trx1 and TrxR1 in more than 50% of NSCLC patients are higher than the 75% quantile expression level the normal samples. The data is from TCGA. *P* values were determined by a two-tailed Mann–Whitney test.

(d). Box plots of Trx1 and TrxR1 transcript expression from tissues at different stages of disease progression in patients with LUAD and LUSC patients (the number of patients from each group are indicated in parentheses along the x-axis labels) from TCGA database. *P* values were determined by a two-tailed Mann–Whitney test.

(e). Survival curves (Kaplan-Meier) and the association of higher Trx1($Trx1^{High}$ n=251; $Trx1^{Low}$ n=249) and TrxR1 ($TrxR1^{High}$ n=126; $TrxR1^{Low}$ n=374) expression with poor overall survival in patients with LUAD (Trx1; **** *p* <0.0001; TrxR1; ** *p* =0.0017). The data is from TCGA.

a.

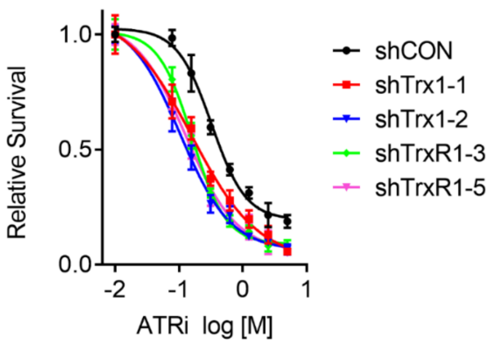

b.

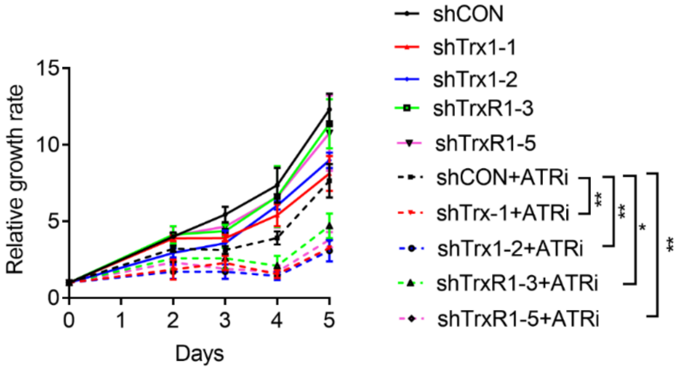

Figure S2. Trx1 or TrxR1 depletion increases the sensitivity of an ATR inhibitor. **(a)** Cell growth after dose-dependent treatment with ATRi (VE821) of H1299 cells knocked down for Trx1 or TrxR1 expression (n=4 biological repeats), error bars represent ± SD. **(b)** The growth rate of H1299 cells treated with an ATRi (1μM) for 24 h and depleted for Trx1 or TrxR1 expression (n= 3 biological repeats; error bars represent ±SD). [Statistical information: Data is presented mean value ±SD; The *p- value*s were calculated using one way ANOVA for multiple comparison; *$p \leq 0.05$; ** $p \leq 0.005$].

a.

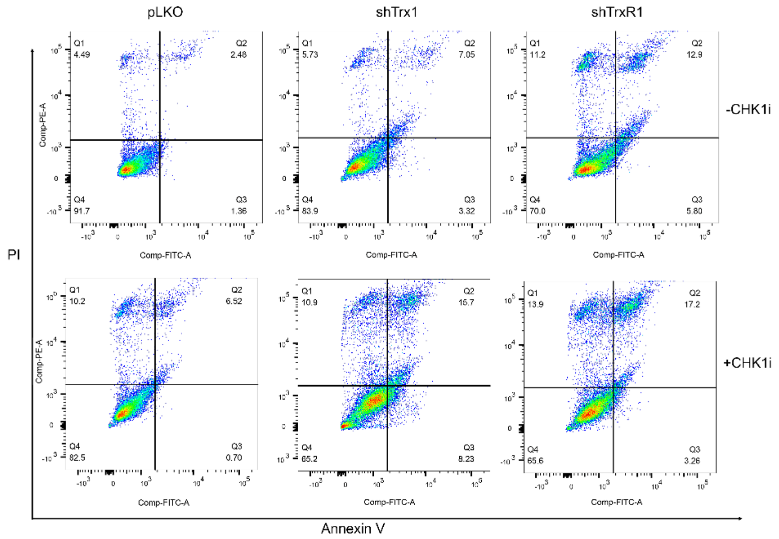

b.

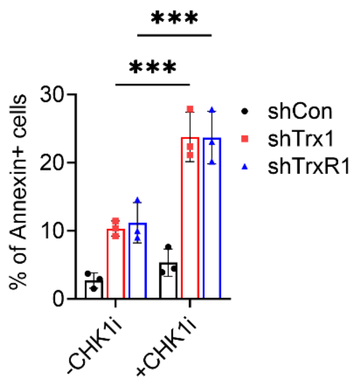

c.

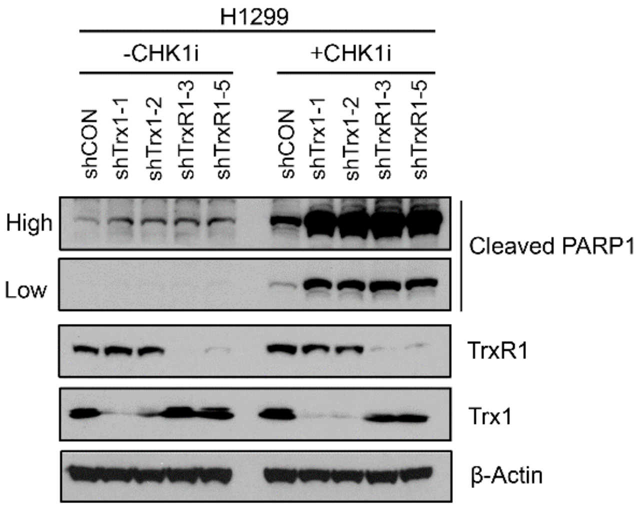

Figure S3. The rate of apoptosis is higher in Trx1 or TrxR1 KD cells treated with CHK1i compared to controls. (a) Flow cytometry of H1299 cells stained with Annexin V and PI to measure apoptosis after treatment with 1μM CHK1i for 24 h following Trx1 or TrxR1 KD. FACS gating strategies are presented in Fig. S17. Dot plot showing distribution of apoptotic/non-apoptotic cells. (**b**) Percent of apoptotic cells (Annexin+) from 3 independent biological replicates; error bars represent ± SD. (**c**) Western blot analysis of the indicated proteins in H1299 cells treated with 1μM CHK1i for 24 h following Trx1 or TrxR1 KD.

[Statistical information: Data are represented as mean value ± SD. The *p- value*s were calculated using one way ANOVA for multiple comparison; *** *p ≤0.001*].

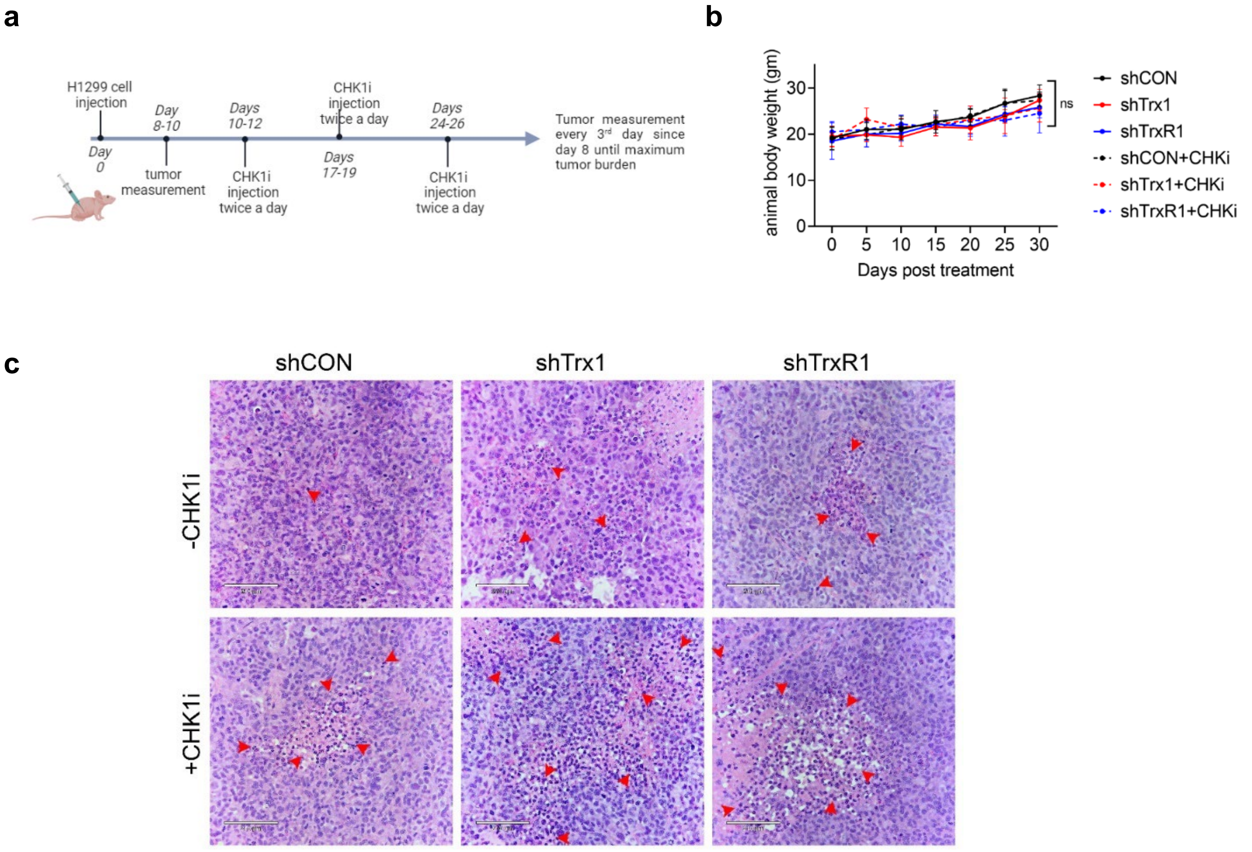

Figure S4. A synergistic interaction between Trx1 or TrxR1 KD and CHKi in NSCLC xenograft with no obvious toxicity. (**a**) A schematic diagram *(Created with BioRender.com released under a Creative Commons Attribution-NonCommercial-NoDerivs 4.0 International license)* illustrating the scheme of the xenograft model and CHK1i administration. (**b**) Mouse body weight is similar among the experimental groups from Figure 2. Effect of CHK1i treatment on overall body weight of animals in the indicated groups. [Statistical information: Data are represented as mean value ± SD; error bars represent ± SD. The *p- value*s were calculated using one way ANOVA for multiple comparison; *ns: non-significant*] (**c**) Representative H&E-stained sections of tumors excised from the indicated groups of animals. Apoptotic and/or necrotic cells/area are indicated by red arow. Scale bar 100µm.

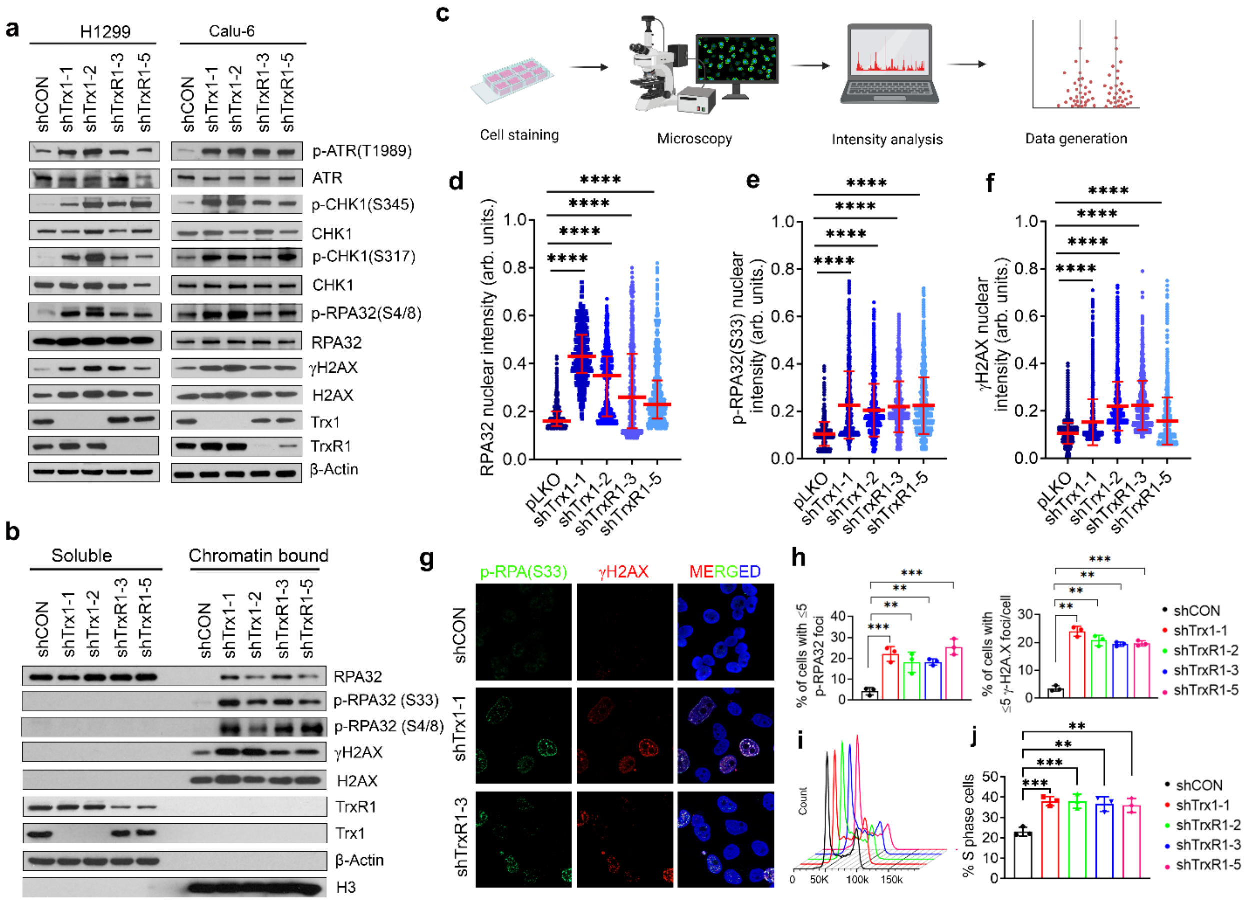
a
H1299
Calu-6
shCON
shTrx1-1
shTrx1-2
shTrxR1-3
shTrxR1-5
p-ATR(T1989)
ATR
p-CHK1(S345)
CHK1
p-CHK1(S317)
CHK1
p-RPA32(S4/8)
RPA32
γH2AX
H2AX
Trx1
TrxR1
β-Actin
b
Soluble
Chromatin bound
RPA32
p-RPA32 (S33)
p-RPA32 (S4/8)
γH2AX
H2AX
TrxR1
Trx1
β-Actin
H3
c
Cell staining
Microscopy
Intensity analysis
Data generation
d
RPA32 nuclear intensity (arb. units.)
e
p-RPA32(S33) nuclear intensity (arb. units.)
f
γH2AX nuclear intensity (arb. units.)
pLKO
****
g
p-RPA(S33)
γH2AX
MERGED
h
% of cells with ≤5 p-RPA32 foci
% of cells with ≤5 γ-H2A.X foci/cell
shTrxR1-2
i
Count
0
50K
100k
150k
j
% S phase cells

k.

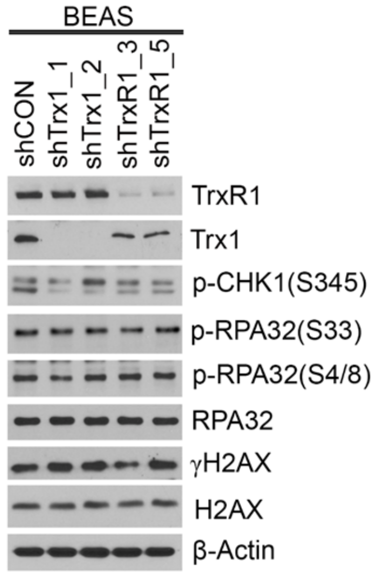
BEAS
shCON
shTrx1_1
shTrx1_2
shTrxR1_3
shTrxR1_5
TrxR1
Trx1
p-CHK1(S345)
p-RPA32(S33)
p-RPA32(S4/8)
RPA32
γH2AX
H2AX
β-Actin

Figure S5. Trx1 or TrxR1 depletion increases replication stress (RS). (**a**). Representative western blots of RS marker proteins in H1299 and Calu-6 cells depleted for Trx1 or TrxR1. (**b**) Representative western blots of the DNA-binding protein RPA32 and phosphorylated RPA32(p-RPA32 S33 and S4/8) and γH2AX in H1299 cells with Trx1 or TrxR1 depletion. Cytoplasmic and chromatin fractions were fractionated using mild and stringent detergent-based buffers respectively. (**c**) An illustration of the scheme *(Created with BioRender.com released under a Creative Commons Attribution-NonCommercial-NoDerivs 4.0 International license)* to measure nuclear intensity of RS markers. (**d-f**) The nuclear intensity of RPA32, p-RPA32(S33) and γH2AX in H1299 cells with Trx1 or TrxR1 depletion (n= ≥1000 cells; error bars represent ± SD). (**g, h**) Representative immunofluorescence images of p-RPA32(S33) and γH2AX staining in the indicated groups. Bar graphs shows the frequency of cells with ≥5 foci/cell in the indicated groups (n=3 biological repeats; error bars represent ± SD), Scale bar 50μm. (**i, j**) The degree of accumulation of cells in the S phase upon Trx1 or TrxR1 depletion by flow cytometric profile of cell cycle progression in the indicated groups. FACS gating strategies are presented in Fig. S17. (i) and its quantitation (n=3 biological repeats; error bars represent ± SD) (j). (**k**) Representative western blots of RS-related proteins in untransformed human normal epithelia BEAS cells upon Trx1 or TrxR1 depletion.

[Statistical information: Data are represented as mean value ± SD. The *p- value*s were calculated using one way ANOVA for multiple comparison. Red line in dot plot (d, e, f) indicates mean; ** *p≤0.005; *** p ≤0.001; **** p ≤0.0001*].

a.

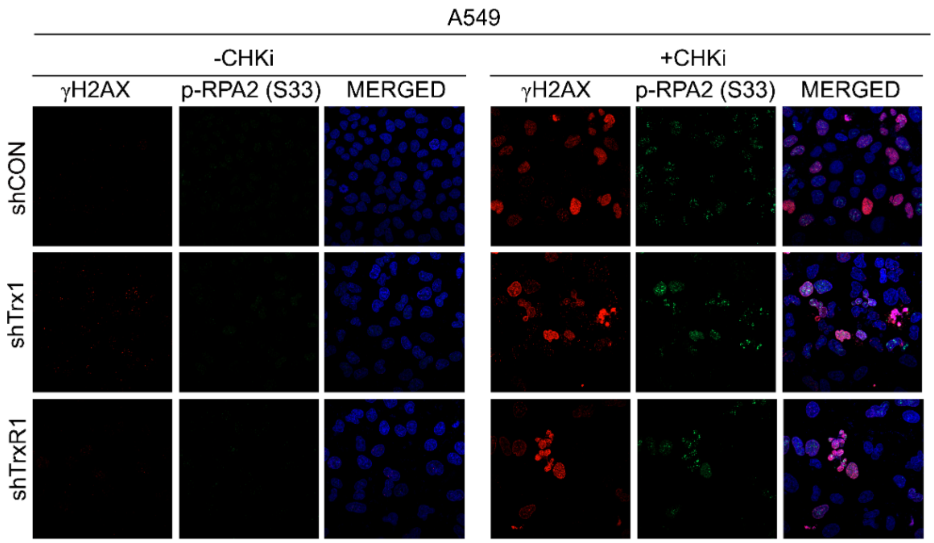
A549
-CHKi
+CHKi
γH2AX
p-RPA2 (S33)
MERGED
γH2AX
p-RPA2 (S33)
MERGED
shCON
shTrx1
shTrxR1

b.

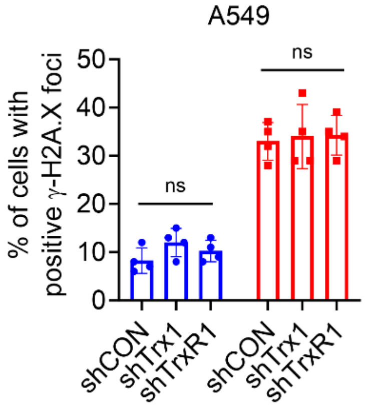
A549
% of cells with positive γ-H2A.X foci
0
10
20
30
40
50
ns
ns
shCON
shTrx1
shTrxR1
shCON
shTrx1
shTrxR1

c.

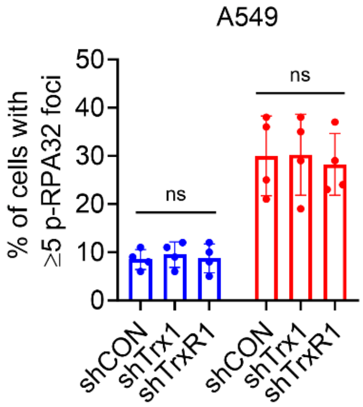
A549
% of cells with ≥5 p-RPA32 foci
0
10
20
30
40
50
ns
ns
shCON
shTrx1
shTrxR1
shCON
shTrx1
shTrxR1

d.

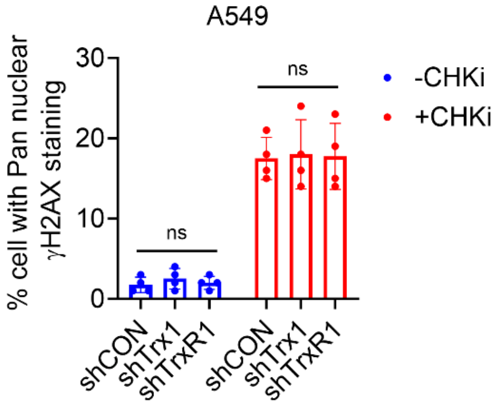
A549
% cell with Pan nuclear γH2AX staining
0
10
20
30
ns
ns
shCON
shTrx1
shTrxR1
shCON
shTrx1
shTrxR1
-CHKi
+CHKi

e.

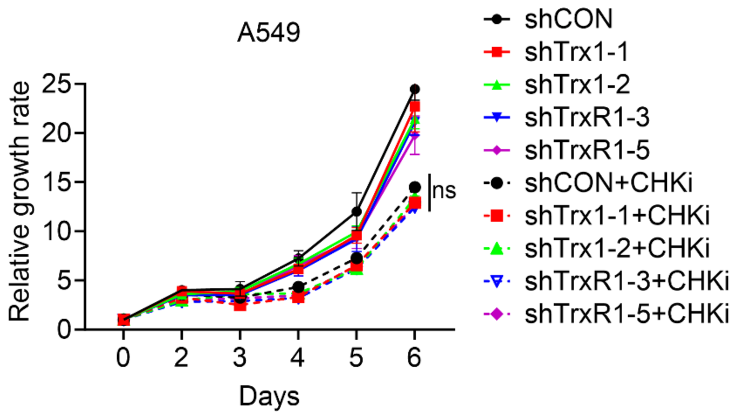
A549
Relative growth rate
0
5
10
15
20
25
0
2
3
4
5
6
Days
ns
shCON
shTrx1-1
shTrx1-2
shTrxR1-3
shTrxR1-5
shCON+CHKi
shTrx1-1+CHKi
shTrx1-2+CHKi
shTrxR1-3+CHKi
shTrxR1-5+CHKi

Figure. S6. Trx1/TrxR1 KD has no significant impact on RS in GSH proficient A549 NSCLC cells. (**a**). Representative immunofluorescence image of p-RPA32 and γH2AX in A549 cells with or without CHKi treatment following Trx1 or TrxR1 KD. (**b-d**). Bar graph showing the percentage of cells with γH2AX**,** p-RPA32(S33) foci and PAN γH2AX staining respectively in A549 cells treated with CHK1i following trx1 or TrxR1 KD (n= 2 biological repeats in duplicates). (**e**). Line graph showing the effect of Trx1 or TrxR1 KD on cell proliferation of A549 cells with or without CHK1i (n= 2 biological repeats in triplicates)

[Statistical information: Data are represented as mean value ± SD; all error bars represent ± SD. The *p- value*s were calculated using one way ANOVA for multiple comparison. ns= Not significant].

a.

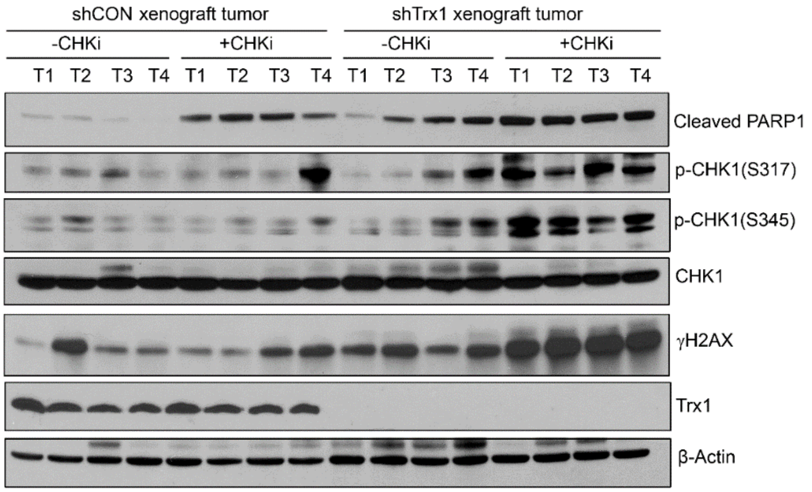

b.

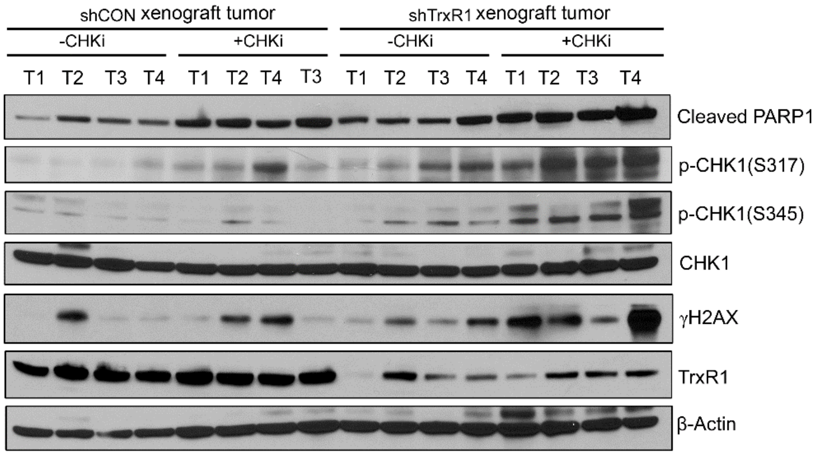

Figure S7. Trx1/TrxR1 KD in combination with CHK1i leads to a greater extent of RS, DNA damage and apoptosis *in vivo*. (**a, b**) More profound level of RS and DNA damage were observed in the xenograft tumor tissue derived from the tumors derived from Trx1 or TrxR1 KD cell treatment with CHKi, as comparison with the tumor tissue derived from the group with either Trx1/TrxR1 KD or CHK1i. Total protein were isolated from randomly selected tumors among the indicated experimental groups. RS and apoptotic marker were detected in Trx1 KD (**a**) group treated with or without CHK1i and TrxR1 KD (**b**) treated with or without CHK1i. T1-T4 are the number of individual tumor tissues.

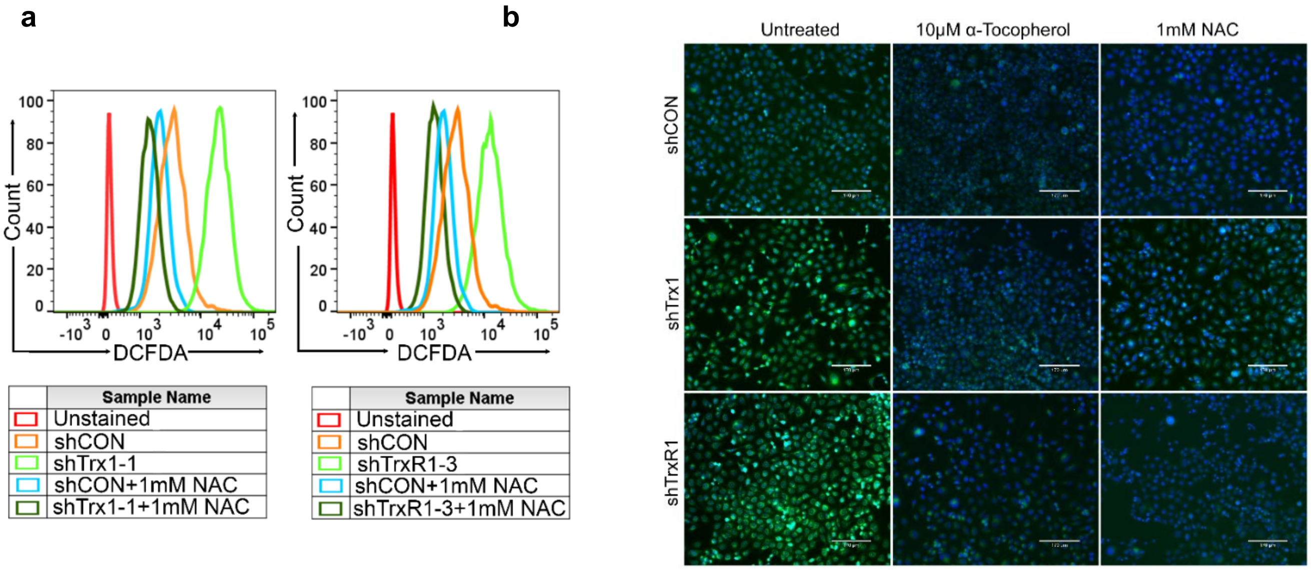

Figure S8. The detection of Trx1 or TrxR1 KD-induced ROS. (a). Intracellular ROS levels in H1299 cells treated with 1 mM NAC as measured by DCFDA staining via flowcytometry. FACS gating strategies are presented in Fig. S17. **(b).** Representative images of lipid peroxidation in Trx1 or TrxR1 KD H1299 cells treated as indicated. All groups of cells were then treated with linoleamide alkyne (LAA) reagent (alkyne-modified linoleic acid) for detection of lipid peroxidation-derived protein modifications in cells. NAC and α-tocopherol were added along with LAA. Cells were fix and analyzed following click-iT reaction.

a.

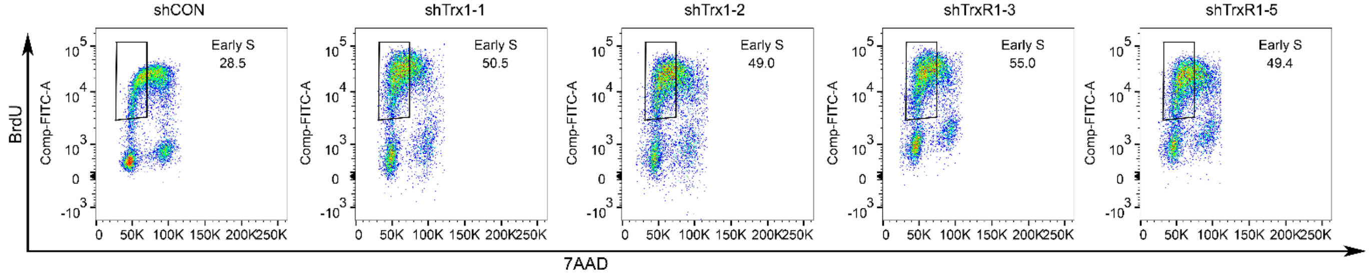

b.

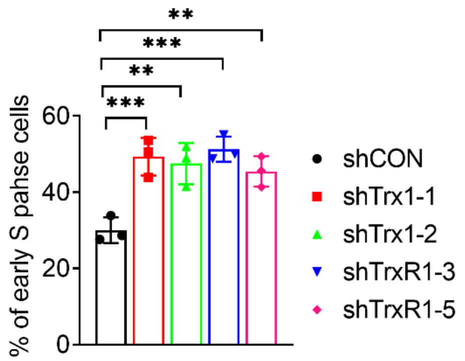

Figure S9. Trx1 or TrxR1 depletion leads to the accumulation of cells in the early S phase. (**a**). The representative dot plot of measurement of BrdU uptake by flow cytometry in actively proliferating cells in the indicated groups. FACS gating strategies are presented in Fig. S17. (**b**) Percent of cells in early S phase upon Trx1 or TrxR1 depletion. Bar graph shows the quantification of flow cytometric data from Figure S9a (n=3 biological repeat; error bars represent ± SD).

[Statistical information: Data are presented as mean value ± SD. The *p- value*s were calculated using one way ANOVA for multiple comparison. ** $p \leq 0.005$; *** $p \leq 0.001$].

a.

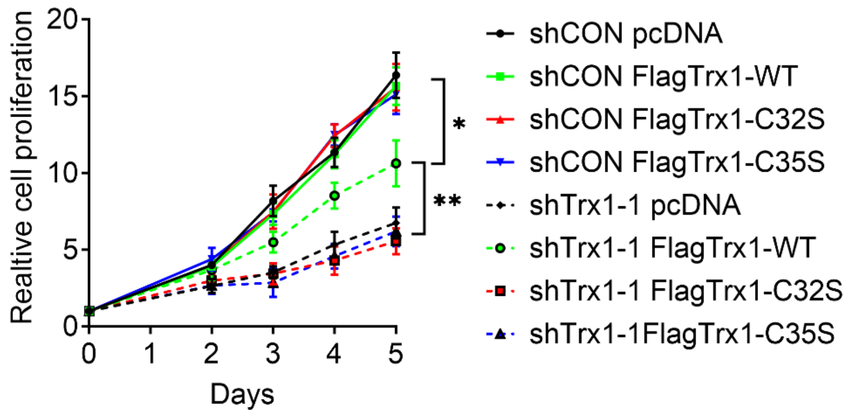

b.

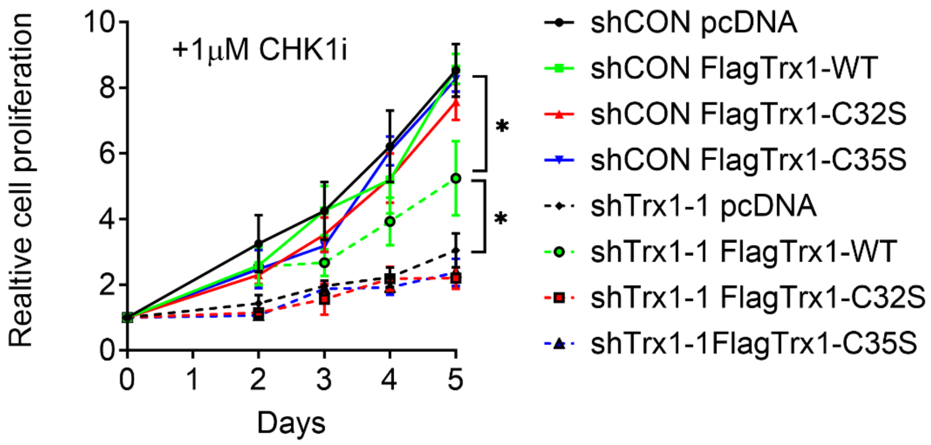

Figure S10. Trx1-WT, but not Trx1 redox mutant expression, partially abrogates Trx1 depletion-induced slow cell proliferation and the sensitivity to CHKi. (**a**) Relative growth of cells expressing Trx1-WT or its redox mutants after Trx1 depletion (n=3 biological repeats). (**b**) Relative growth of cells expressing Trx1-WT or its redox mutants in the presence of a CHK1i inhibitor with or without Trx1 depletion (n=3 biological repeats).

[Statistical information: Data are presented as mean value ± SD; all error bars represent ± SD. The *p- value*s were calculated using one way ANOVA for multiple comparison; **p*≤0.05; ** *p≤0.005*]

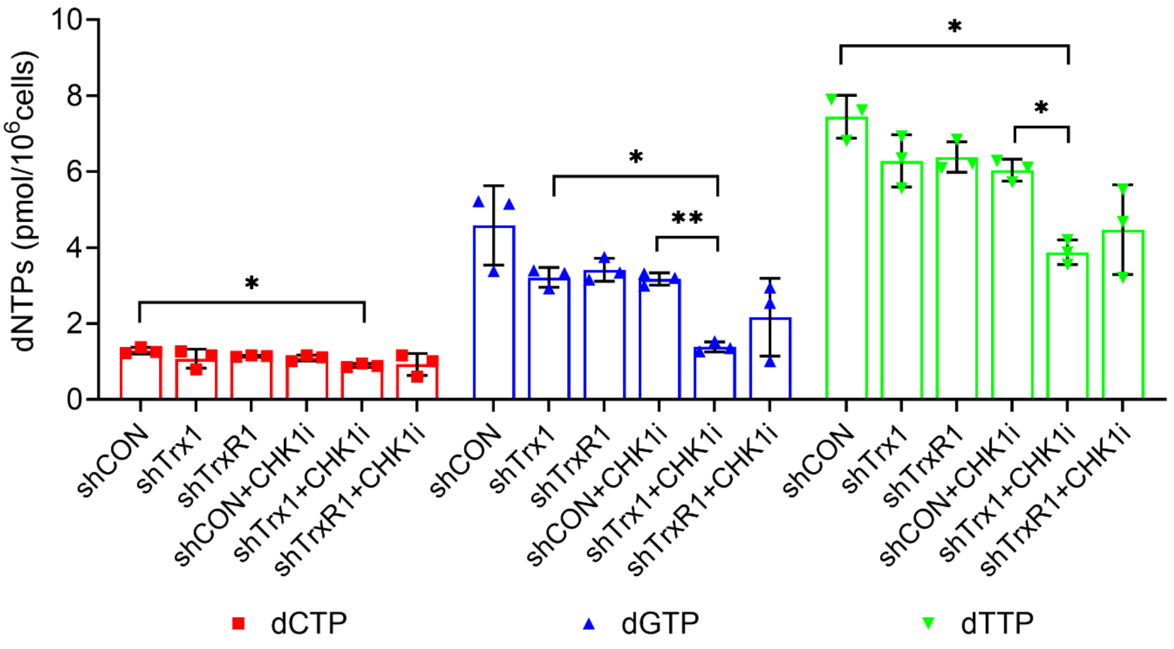

Figure S11. Levels of the indicated dNTPs in cells with Trx1 or TrxR1 depletion with or without in combination of CHK1 inhibition. Total cellular dNTPs were subjected to RT-based primer extension assay to quantify each dCTP, dGTP and dTTP concentration (n=3 biological repeats).

[Statistical information: Data are presented as mean value ± SD; all error bars represent ± SD. The *p- value*s were calculated using one way ANOVA for multiple comparison; *$p \leq 0.05$; ** $p \leq 0.005$]

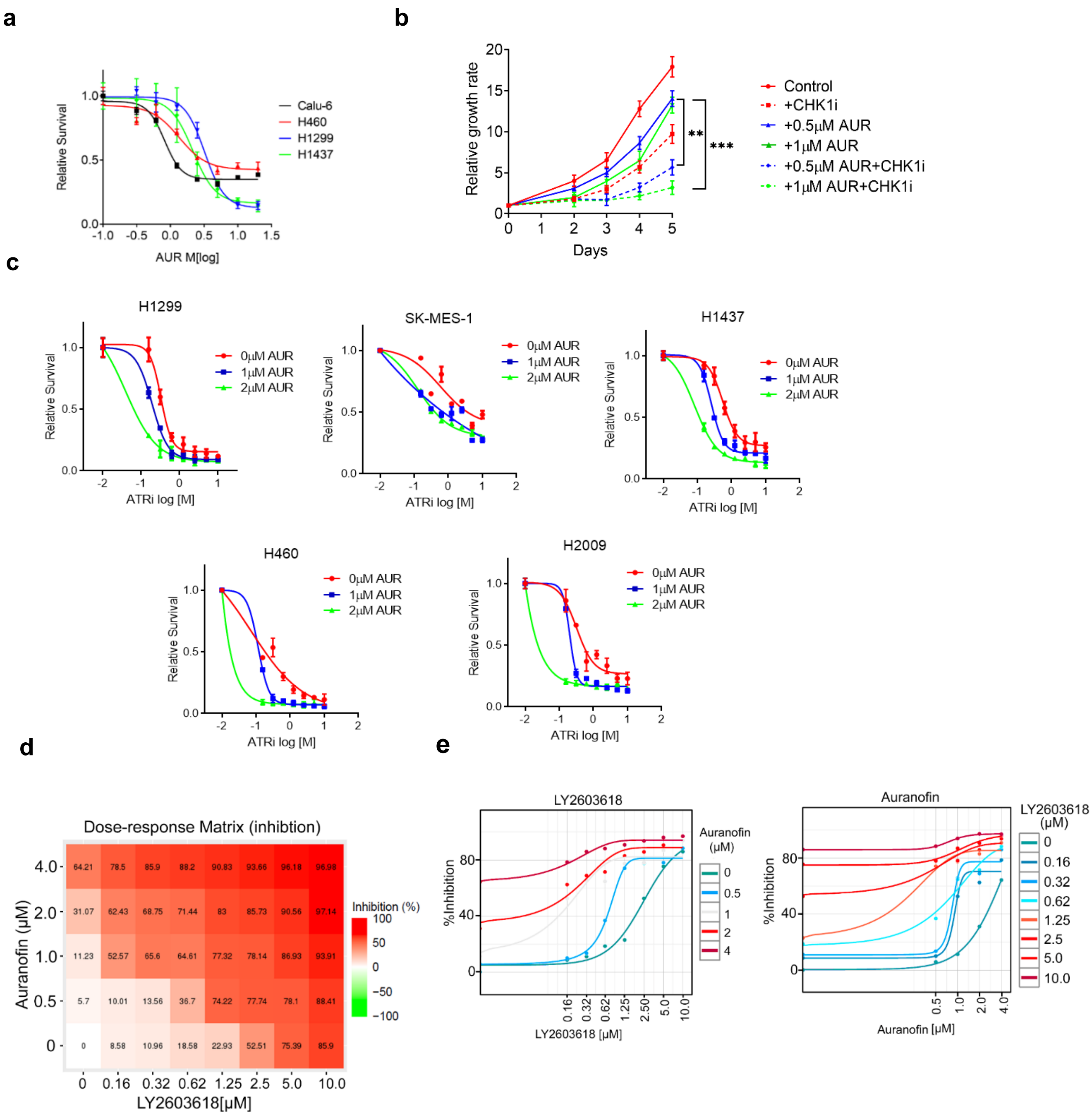

Figure S12. The synergistic interaction of AUR and CHK1i or ATRi. (a) The relative survival of different NSCLC cell lines treated with AUR in a dose dependent manner (n=3 biological repeats) **(b)** Relative cell proliferation of H1299 cells treated with the indicated doses of CHK1i, AUR or AUR + CHK1i for 24 h. Cell proliferation was accessed at indicated time points (n=3 biological repeats). **(c)** The relative survival of different NSCLC cell lines treated with ATRi and AUR (n=3 biological repeats) **(d)** A dose-response matrix generated via *SynergyFinder 2.0* at different concentration of AUR and CHK1i. **(e)** Synergistic Potency shift between CHKi and AUR generated via *SynergyFinder 2.0*. The percent inhibition of cell growth to measure the potency shift between AUR and CHK1i. Synergistic potency shift was induced 34752.01 times by AUR

towards CHK1i. CHKi induced 7.25 times synergistic potency shift towards AUR; There was 0.15 times negative cooperativity between AUR and CHKi.

[Statistical information: Data are presented as mean value ± SD; all error bars represent ± SD. The *p- value*s were calculated using one way ANOVA for multiple comparison; ** $p \leq 0.005$; *** $p \leq 0.001$]

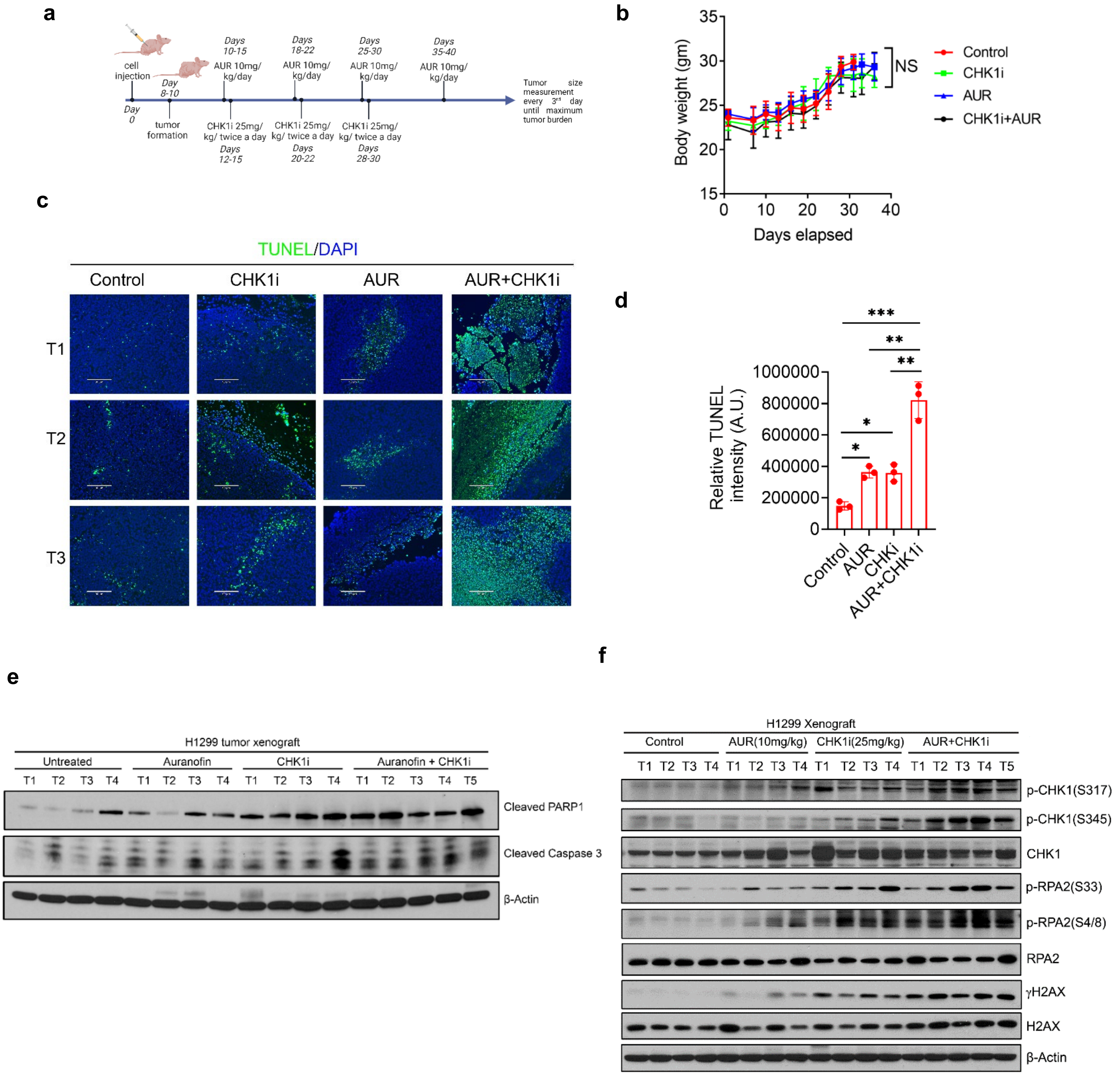

a
cell injection
Day 0
Day 8-10
tumor formation
Days 10-15
AUR 10mg/ kg/day
Days 18-22
AUR 10mg/ kg/day
Days 25-30
AUR 10mg/ kg/day
Days 35-40
AUR 10mg/ kg/day
CHK1i 25mg/ kg/ twice a day
Days 12-15
CHK1i 25mg/ kg/ twice a day
Days 20-22
CHK1i 25mg/ kg/ twice a day
Days 28-30
Tumor size measurement every 3rd day until maximum tumor burden
b
Body weight (gm)
Days elapsed
NS
Control
CHK1i
AUR
CHK1i+AUR
c
TUNEL/DAPI
Control
CHK1i
AUR
AUR+CHK1i
T1
T2
T3
d
Relative TUNEL intensity (A.U.)
Control
AUR
CHKi
AUR+CHK1i
e
H1299 tumor xenograft
Untreated
Auranofin
CHK1i
Auranofin + CHK1i
T1 T2 T3 T4 T1 T2 T3 T4 T1 T2 T3 T4 T1 T2 T3 T4 T5
Cleaved PARP1
Cleaved Caspase 3
β-Actin
f
H1299 Xenograft
Control
AUR(10mg/kg)
CHK1i(25mg/kg)
AUR+CHK1i
T1 T2 T3 T4 T1 T2 T3 T4 T1 T2 T3 T4 T1 T2 T3 T4 T5
p-CHK1(S317)
p-CHK1(S345)
CHK1
p-RPA2(S33)
p-RPA2(S4/8)
RPA2
γH2AX
H2AX
β-Actin

Figure S13. A synergy between AUR and CHKi in treating NSCLC xenograft without observed toxicity. (a) Graphical representation *(Created with BioRender.com released under a Creative Commons Attribution-NonCommercial-NoDerivs 4.0 International license)* of the scheme of drug administration in the xenograft model. **(b)** Body weights of animals treated with indicated treatment groups over the course of treatment duration (n=5 animals in each group; one way ANOVA was used for multiple comparison; data are presented as mean value ± SD; error bars represent ± SD). **(c)** Representative tumor tissues sections indicating TUNEL staining in each treatment group in H1299 xenograft treated with indicated inhibitor group. T1-T3 are the number of tumors. Scale bar 100μm. **(d)** Bar graph showing relative intensities of TUNEL staining in each treatment groups (n=3 individual tumors form each group; one way ANOVA was used to calculate the *p* value for multiple comparison; data are presented as mean value ± SD; error bars represent ± SD). **(e)** Representative WB showing level of cleaved PARP1 and cleaved caspase 3 in randomly selected tumor tissue in indicated treatment groups. T1-T5 are the number of tumors. **(f)** Representative WB showing level of RS and DNA damage marker in randomly selected tumor tissue in indicated treatment groups. T1-T5 are the number of tumors.

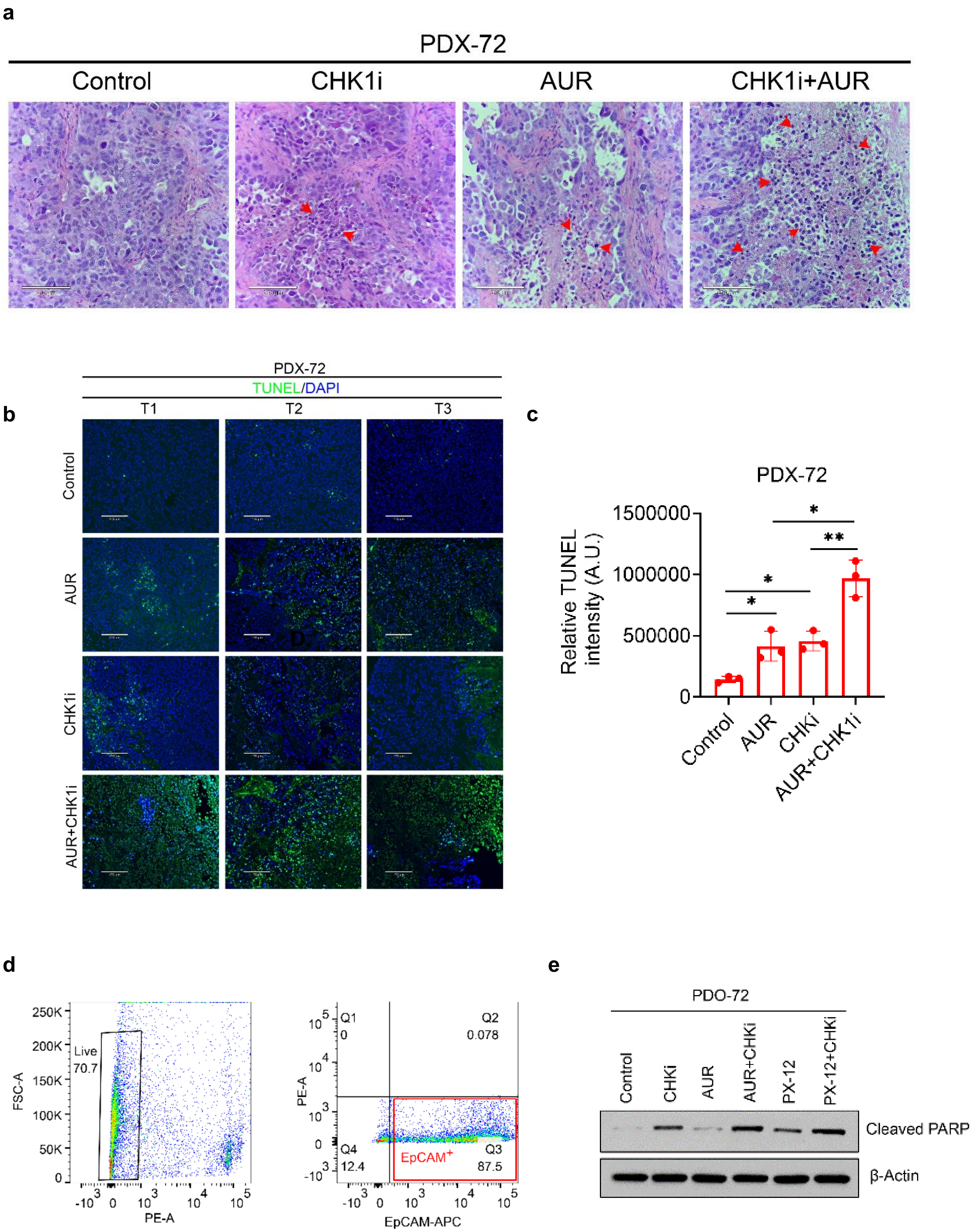
a
PDX-72
Control
CHK1i
AUR
CHK1i+AUR
b
PDX-72
TUNEL/DAPI
T1
T2
T3
Control
AUR
CHK1i
AUR+CHK1i
c
PDX-72
Relative TUNEL intensity (A.U.)
1500000
1000000
500000
0
*
**
*
*
Control
AUR
CHKi
AUR+CHK1i
d
250K
200K
150K
100K
50K
0
FSC-A
Live
70.7
PE-A
EpCAM-APC
Q1
0
Q2
0.078
Q4
12.4
Q3
87.5
EpCAM+
e
PDO-72
Control
CHKi
AUR
AUR+CHKi
PX-12
PX-12+CHKi
Cleaved PARP
β-Actin

Figure S14. AUR has a synergy with CHK1i in promotion of cell death uisng PDX and PDO models. (a) Representative H&E-stained sections of tumors excised from indicated groups PDX-72 treatment group. Red arrows indicating apoptotic and/or necrotic cells. Scale bar 50μm **(b)** Representative tumor tissues sections indicating TUNEL staining in each treatment group in PDX-72 tumor model. T1-T3 are the number of tumors. Scale bar 100 μm**. (c)** Bar graph showing relative intensities of TUNEL staining in each treatment groups (n=3 from each group; one way ANOVA was used to calculate the p value for multiple comparison; data are presented as mean value ± SD; error bars represent ± SD.). **(d)** PDX-72 untreated tumors were dissociated using tumor dissociation kit and cancer cells were isolated using human cancer cell isolation kit. Human cancer cells were further enriched by depletion of mouse cell using via mouse cell depletion kit. Next, isolated cells were stained with Human-EpCAM (CD326) to verify the isolation of human cancer cells. Indicated flowcytometric panel indicating the ≥87% live human cancer cells derived from PDX-72 tumors. FACS gating strategies are presented in Fig. S17. Isolated human cancers were used for organoid generations. (**e**). WB blot indicating level of cleaved PARP1 in PDO-72 treated with indicated inhibitors for 48hrs.

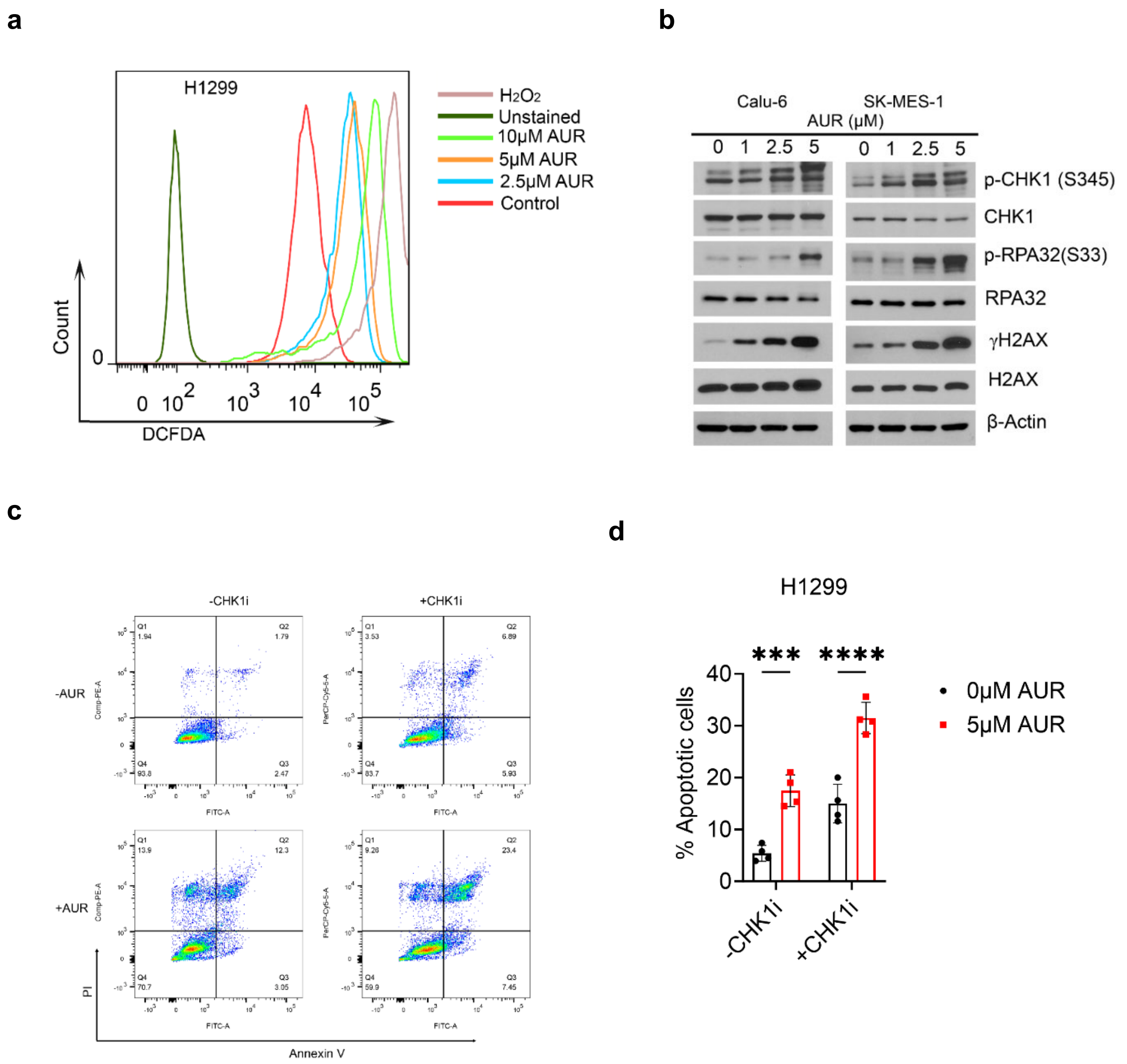

Figure S15. The synergy between AUR and CHK1i in RS and apoptosis. (a) Flow cytometric profile of DCFDA intensity in the indicated groups. H_2O_2 was used as a control. FACS gating strategies are presented in Fig. S17. **(b)** Representative western blots of RS marker proteins in the indicated NSCLC cell lines treated with AUR. **(c)** Flow cytometry of H1299 cells treated as indicated for 24 hrs followed by staining with Annexin V and PI to assess apoptosis. Dot plot showing distribution of apoptotic/non-apoptotic cells. FACS gating strategies are presented in Fig. S17. (**d**) Percentage of apoptotic cells (Annexin+) cells from 4 independent biological replicates; data are presented as mean value ± SD; error bars represent ± SD. Two tailed student t test was used to calculate the p value. ***≤0.001, ****≤0.0001.

a

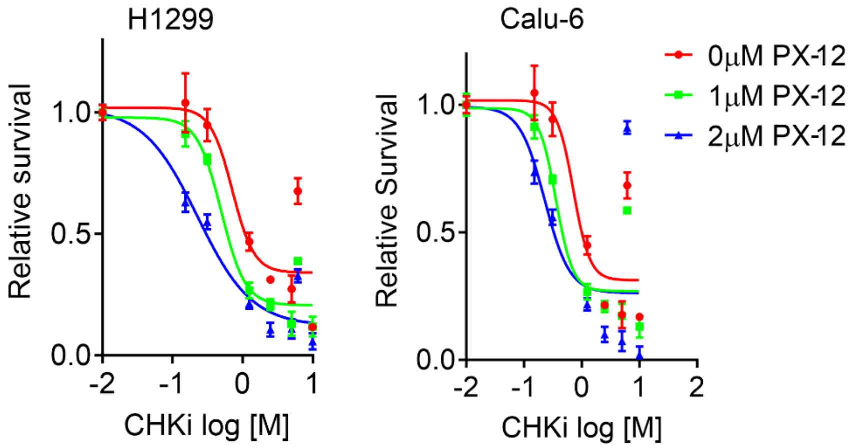

b

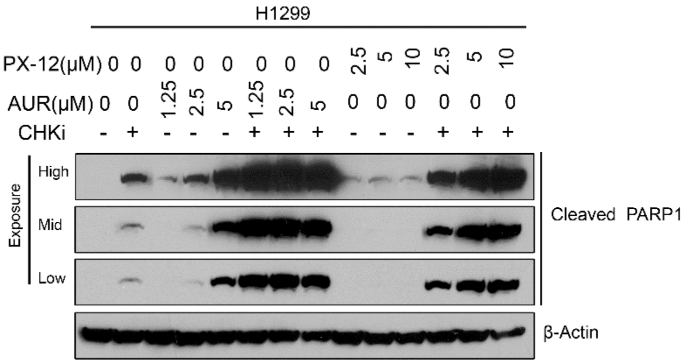

c

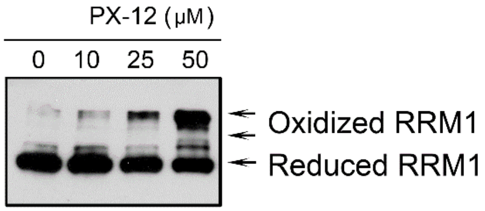

Figure S16. Synergy and profound apoptosis occurrence in the cells treated with the combined PX12- and CHK1i. (a) The cytotoxicity of combined of PX-12 with CHK1i on the indicated NSCLC cell lines in dose dependent manner (n=3 biological repeats), data are presented as mean value ± SD; error bars represent ± SD. **(b)** Assessment of cleaved PARP1, a marker of apoptosis, in H1299 cells treated with the indicated drugs via WB. **(c)** PX-12 treatment enhanced the level of RRM1 oxidation in H1299 cells.

Gating strategies:

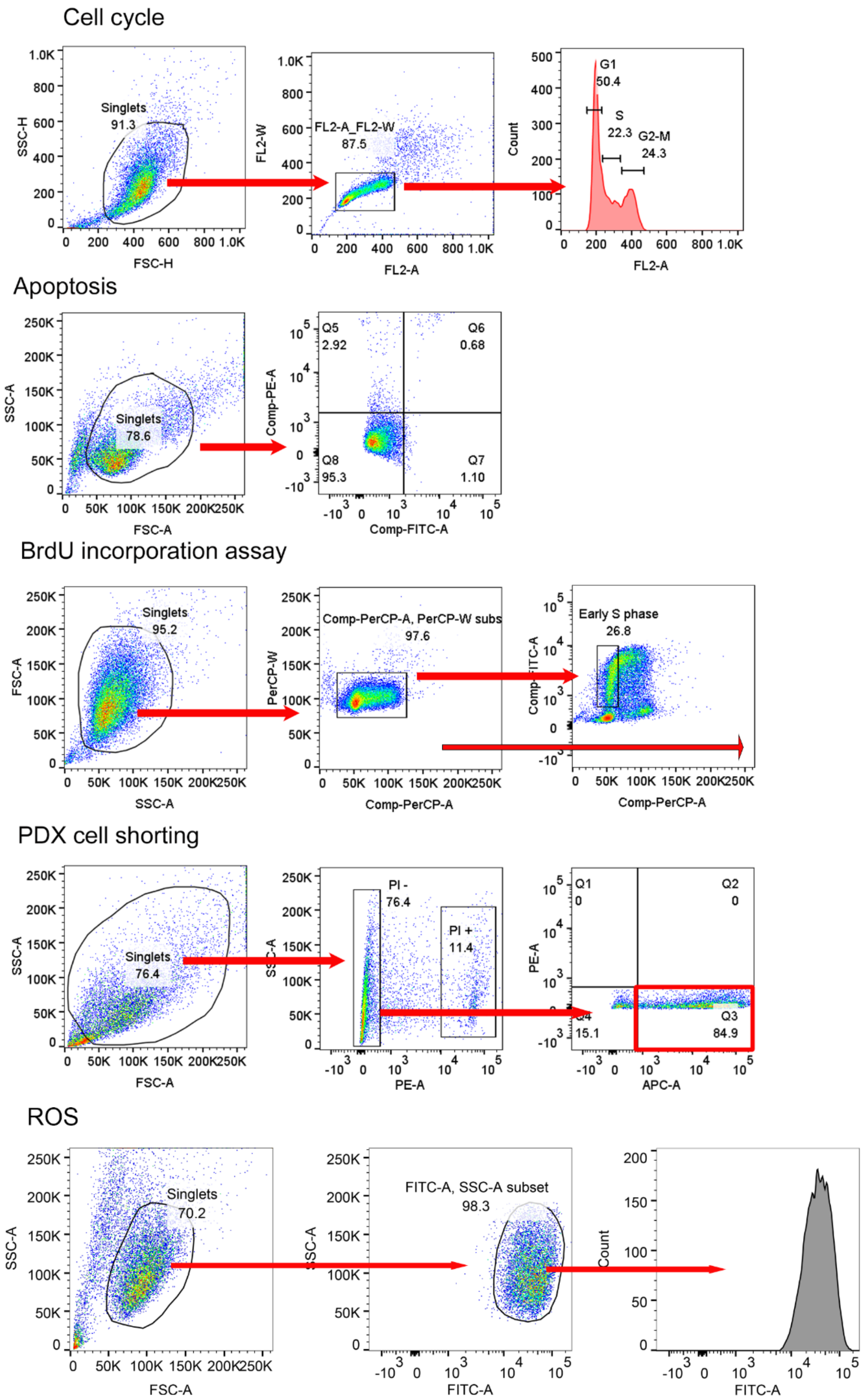

Figure: S17: Scheme showing cell gating strategy in the indicated experiments.

Supplementary Methods.

Hematoxylin and eosin (H&E) and TUNEL assay. Tissue sections were deparaffinized and rehydrated through graded alcohol and mounted in tissue mounting medium following staining with H&E stain. TUNEL assays were performed using the *In Situ* Cell Death Detection Kit (Roche; #11684795910) following manufacturer's protocol.

Lipid peroxidation assay. Lipid peroxidation was detected using Click-iT™ Lipid Peroxidation Imaging Kit (Thermo Fisher Scientific. # Cat. C10446). Briefly, Cells were grown on coverslips in multiwall plates. Cells were then treated with linoleamide alkyne (LAA) reagent (alkyne-modified linoleic acid) for detection of lipid peroxidation-derived protein modifications in cells. NAC and α-tocopherol were added along with LAA. Next, cells were fixed using 4% paraformaldehyde and blocked in 1% BSA following PBS wash in between. Click-It reaction cocktail was added for 30 min in dark for Click-It reaction and cells were washed with PBS subsequently. Cells were counter stained with DAPI and imaged on Echo Revolve fluorescence microscope. Fluorescent intensity of each cell was analyzed using Image J.

Uncropped WB related to WB panels presented in supplementary figures.

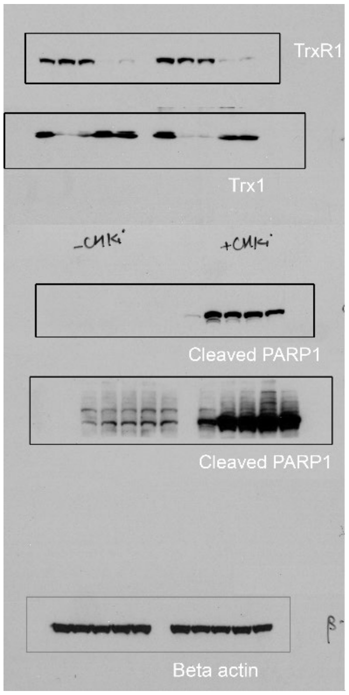

H1299

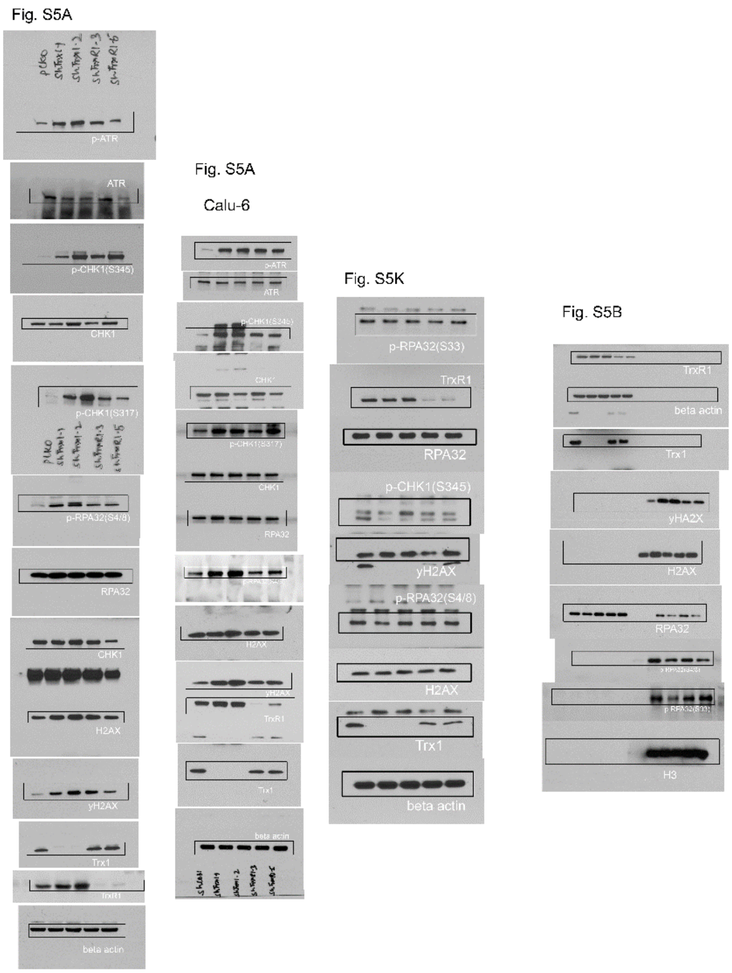

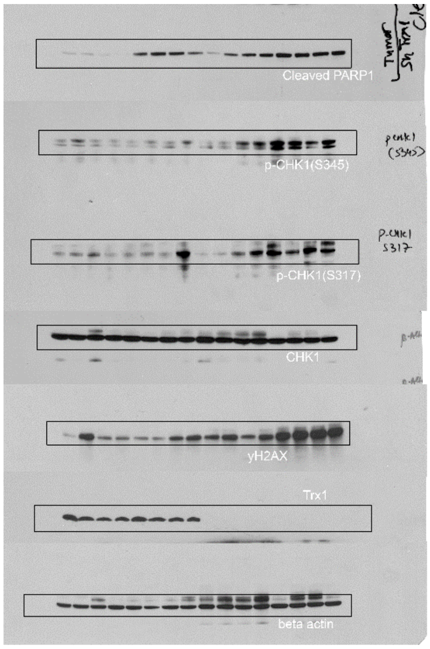

Fig. S7A

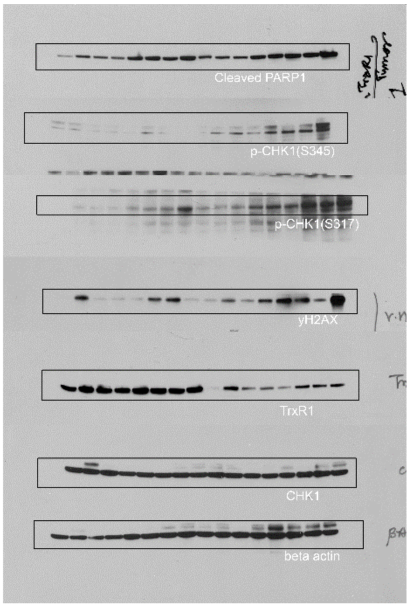

Fig. S7B

Figure. S13E.

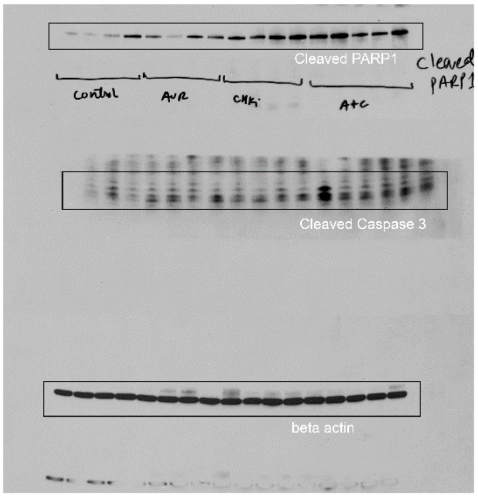

Figure S13F.

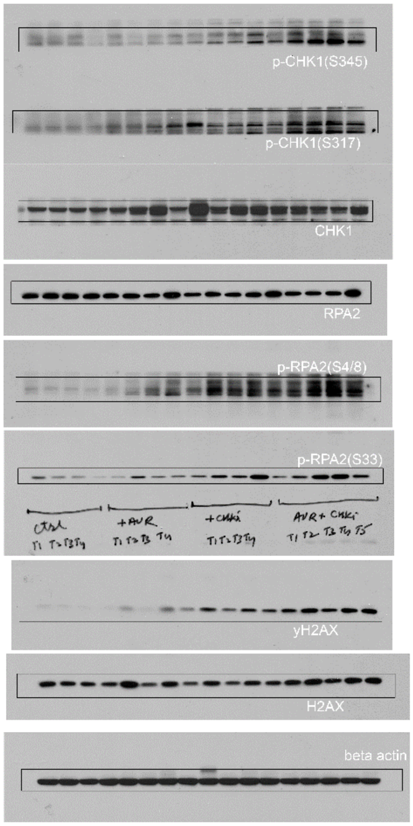

Figure S14e.

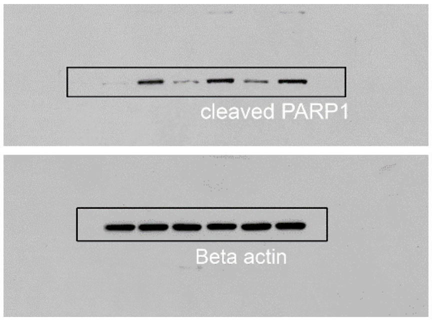

Figure. S15B

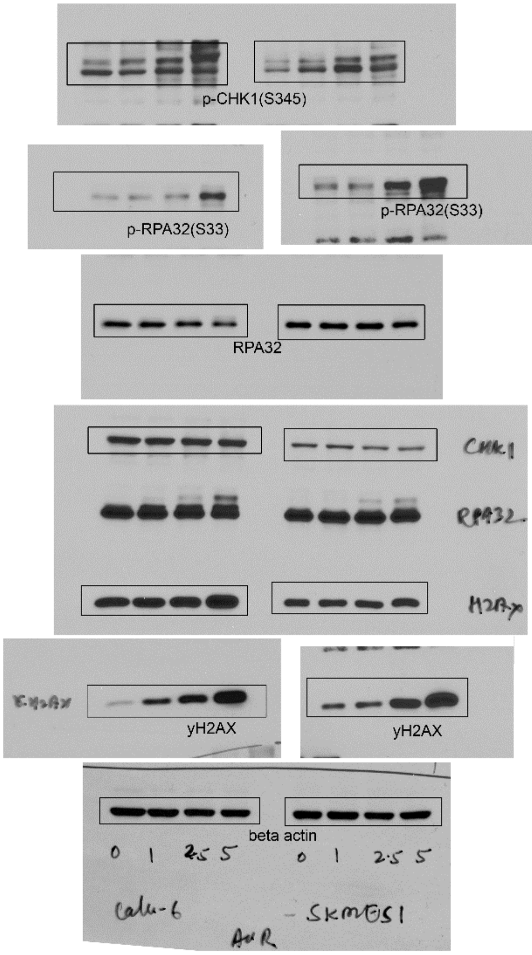

Figure S16.

Fig. 16B

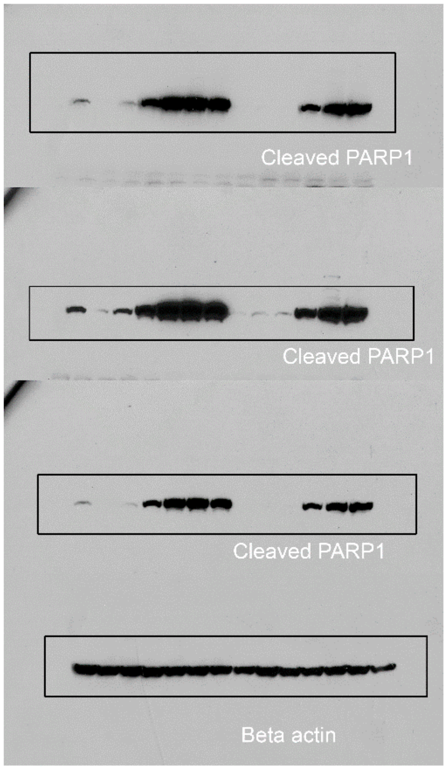

Fig. 16C

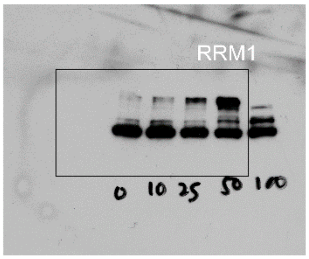

Supplement: Supplementary file 1 — Supplementary Information [file 41467_2024_48076_MOESM1_ESM.pdf]
