# Supplementary material for: The thioredoxin system determines CHK1 inhibitor sensitivity via redox-mediated regulation of ribonucleotide reductase activity
Source: Nat Commun. 2024 May 31;15:4667. doi: 10.1038/s41467-024-48076-9 (PMC11143221; doi:10.1038/s41467-024-48076-9)
Supplement: Supplementary file 2 — Reporting Summary [file 41467_2024_48076_MOESM2_ESM.pdf]

nature portfolio

Corresponding author(s): Junran Zhang

Last updated by author(s): 2/22/2024

Reporting Summary

Nature Portfolio wishes to improve the reproducibility of the work that we publish. This form provides structure for consistency and transparency in reporting. For further information on Nature Portfolio policies, see our Editorial Policies and the Editorial Policy Checklist.

Statistics

For all statistical analyses, confirm that the following items are present in the figure legend, table legend, main text, or Methods section.

n/a	Confirmed	
[ ]	[x]	The exact sample size (*n*) for each experimental group/condition, given as a discrete number and unit of measurement
[ ]	[x]	A statement on whether measurements were taken from distinct samples or whether the same sample was measured repeatedly
[ ]	[x]	The statistical test(s) used AND whether they are one- or two-sided<br>*Only common tests should be described solely by name; describe more complex techniques in the Methods section.*
[x]	[ ]	A description of all covariates tested
[x]	[ ]	A description of any assumptions or corrections, such as tests of normality and adjustment for multiple comparisons
[ ]	[x]	A full description of the statistical parameters including central tendency (e.g. means) or other basic estimates (e.g. regression coefficient) AND variation (e.g. standard deviation) or associated estimates of uncertainty (e.g. confidence intervals)
[ ]	[x]	For null hypothesis testing, the test statistic (e.g. *F*, *t*, *r*) with confidence intervals, effect sizes, degrees of freedom and *P* value noted<br>*Give P values as exact values whenever suitable.*
[x]	[ ]	For Bayesian analysis, information on the choice of priors and Markov chain Monte Carlo settings
[x]	[ ]	For hierarchical and complex designs, identification of the appropriate level for tests and full reporting of outcomes
[x]	[ ]	Estimates of effect sizes (e.g. Cohen's *d*, Pearson's *r*), indicating how they were calculated

Our web collection on statistics for biologists contains articles on many of the points above.

Software and code

Policy information about availability of computer code

Data collection	No software/tools/algorithms/packages were used to collect data in this study.
Data analysis	No custom software/tools/algorithms/packages were used to collect data in this study. All mentioned data/statistical analysis was calculated using GraphPad Prism version 10.0

For manuscripts utilizing custom algorithms or software that are central to the research but not yet described in published literature, software must be made available to editors and reviewers. We strongly encourage code deposition in a community repository (e.g. GitHub). See the Nature Portfolio guidelines for submitting code & software for further information.

Data

Policy information about availability of data

All manuscripts must include a data availability statement. This statement should provide the following information, where applicable:

- Accession codes, unique identifiers, or web links for publicly available datasets
- A description of any restrictions on data availability
- For clinical datasets or third party data, please ensure that the statement adheres to our policy

The authors declare that all the other data supporting the findings of this study are available within the article and its supplementary information files. Following data bases were used to procure patient datasets:
https://www.cbioportal.org/

https://ualcan.path.uab.edu/index.html
https://www.oncomine.org

Research involving human participants, their data, or biological material

Policy information about studies with human participants or human data. See also policy information about sex, gender (identity/presentation), and sexual orientation and race, ethnicity and racism.

Reporting on sex and gender	N/A
Reporting on race, ethnicity, or other socially relevant groupings	N/A
Population characteristics	N/A
Recruitment	N/A
Ethics oversight	N/A

Note that full information on the approval of the study protocol must also be provided in the manuscript.

Field-specific reporting

Please select the one below that is the best fit for your research. If you are not sure, read the appropriate sections before making your selection.

[x] Life sciences [ ] Behavioural & social sciences [ ] Ecological, evolutionary & environmental sciences

For a reference copy of the document with all sections, see nature.com/documents/nr-reporting-summary-flat.pdf

Life sciences study design

All studies must disclose on these points even when the disclosure is negative.

Sample size	Sample size was determined based on similar studies in this field such as; https://doi.org/10.1158/0008-5472.CAN-20-0057, https://doi.org/10.1136/gutjnl-2021-325851
Data exclusions	In xenograft experiments animals with visible ulcer were excluded from the study
Replication	To insure reproducibility experiments were conducted at least three times independently with similar results unless stated otherwise.
Randomization	For in vivo experiments, mice were randomly allocated into different treatment groups prior to drug injection.
Blinding	dNTP data was conducted in a double blind manner. Investigators were blinded for group allocations.

Reporting for specific materials, systems and methods

We require information from authors about some types of materials, experimental systems and methods used in many studies. Here, indicate whether each material, system or method listed is relevant to your study. If you are not sure if a list item applies to your research, read the appropriate section before selecting a response.

Materials & experimental systems

n/a	Involved in the study	
[ ]	[x]	Antibodies
[ ]	[x]	Eukaryotic cell lines
[x]	[ ]	Palaeontology and archaeology
[ ]	[x]	Animals and other organisms
[x]	[ ]	Clinical data
[x]	[ ]	Dual use research of concern
[x]	[ ]	Plants

Methods

n/a	Involved in the study	
[x]	[ ]	ChIP-seq
[ ]	[x]	Flow cytometry
[x]	[ ]	MRI-based neuroimaging

Antibodies

Antibodies used	Proteins of interest were detected using appropriate primary antibodies. Antibodies (Trx1; #2429 clone-C63C6; TrxR1; #15140 clone-D1T3D; p-CHK1 (S345); #12302 clone-D12H3; p-CHK1 (S317); #8191 clone-D7H2; CHK1; #2360 clone- 2G1D5; p-ATR(T1989); #30632

clone-D5K8W; ATR; #13934 clone-E1S3S; Histone H3; #4499 clone-D1H2; Histone H2AX; #7631 clone-D17A3; RPA32; #2208 clone-4E4 and RRM1; #8637 clone-D12F12; RRM2; #65939 clone-E7Y9J; E2F1; #3742, cleaved PARP1 #5625 clone-D64E10, Cleaved caspase 3; #9661 clone-5A1E) were purchased from Cell Signaling Technologies. The anti-p53R2 antibody was purchased from Santa Cruze Biotechnology (#sc-137174 clone A-5). The anti-β-actin antibody was purchased form Sigma Aldrich cat#A5441. Antibodies to detect p-RPA32(S4/8) (cat# A300-245A) and p-RPA32(S33) (cat# A300-246A) were purchased from Bethyl Laboratories. The anti-γH2AX antibody (cat#05-636) clone-JBW301 was procured from EMD Millipore. Anti-p-RPA32 (S33 and S4/8) antibodies were used at 1:1500 and anti-β-actin was used at 1:5000 dilution. HRP-tagged secondary antibodies (Anti-rabbit IgG (#7074); Anti-mouse IgG (#7076) and Anti-rat IgG (#7077) from Cell signaling technology) at 1:5000 dilution. For immunofluorescence (IF); Chicken anti-Rabbit IgG (H+L) Cross-Adsorbed Secondary Antibody, Alexa Fluor™ 488; Catalog #A-21441, Goat-anti-Mouse-IgG-H-L-Highly-Cross-Adsorbed Secondary Antibody, Alexa Fluor™ 488; Catalog #A32723, Goat-anti-Mouse-IgG-H-L-Highly-Cross-Adsorbed-Secondary-Antibody Alexa Fluor™ 594; Catalog #A-11032, Goat anti-Rat IgG (H+L) Cross-Adsorbed Secondary Antibody, Alexa Fluor™ 488; catalog #A-110006 was purchased from ThermoFisher Scientific. Anti BrdU antibody was procured form BD Biosciences catalog #347580, Clone B44. Anti-BrdU antibody [clone BU1/75 (ICR1)] Catalog #ab6326 was purchased from Abcam. Anti-human-EpCAM (CD326) antibody was purchased from Miltenyi Biotech (Cat3130-111-000; clone REA764).

Validation

https://www.cellsignal.com/products/primary-antibodies/thioredoxin-1-c63c6-rabbit-mab/2429
https://www.cellsignal.com/products/primary-antibodies/trxr1-d1t3d-rabbit-mab/15140
https://www.cellsignal.com/products/primary-antibodies/phospho-chk1-ser317-d12h3-xp-rabbit-mab/12302
https://www.cellsignal.com/products/primary-antibodies/phospho-chk1-ser317-d7h2-rabbit-mab/8191
https://www.cellsignal.com/products/primary-antibodies/chk1-2g1d5-mouse-mab/2360
https://www.cellsignal.com/products/primary-antibodies/phospho-atr-thr1989-d5k8w-rabbit-mab/30632
https://www.cellsignal.com/products/primary-antibodies/atr-e1s3s-rabbit-mab/13934
https://www.cellsignal.com/products/primary-antibodies/histone-h3-d1h2-xp-rabbit-mab/4499
https://www.cellsignal.com/products/primary-antibodies/histone-h2a-x-d17a3-xp-rabbit-mab/7631
https://www.cellsignal.com/products/primary-antibodies/rpa32-rpa2-4e4-rat-mab/2208
https://www.cellsignal.com/products/primary-antibodies/rrm1-d12f12-xp-rabbit-mab/8637
https://www.cellsignal.com/products/primary-antibodies/rrm2-e7y9j-xp-rabbit-mab/65939
https://www.cellsignal.com/products/primary-antibodies/cleaved-parp-asp214-d64e10-xp-174-rabbit-mab/5625
https://www.cellsignal.com/products/primary-antibodies/cleaved-caspase-3-asp175-antibody/9661
https://www.scbt.com/p/p53r2-antibody-a-5
https://www.sigmaaldrich.com/US/en/product/sigma/a5441
https://www.fortislife.com/products/primary-antibodies/rabbit-anti-phospho-rpa32-s4-s8-antibody/BETHYL-A300-245
https://www.fortislife.com/products/primary-antibodies/rabbit-anti-phospho-rpa32-s33-antibody/BETHYL-A300-246
https://www.bdbiosciences.com/en-us/products/reagents/flow-cytometry-reagents/clinical-discovery-research/single-color-antibodies-ruo-gmp/purified-mouse-anti-brdu.347580
https://www.abcam.com/products/primary-antibodies/brdu-antibody-bu175-icr1-proliferation-marker-ab6326.html
https://www.miltenyibiotec.com/US-en/products/cd326-epcam-antibody-anti-human-reafinity-rea764.html#conjugate=apc

Secondary antibodies
https://www.cellsignal.com/products/secondary-antibodies/anti-rabbit-igg-hrp-linked-antibody/7074
https://www.cellsignal.com/products/secondary-antibodies/anti-mouse-igg-hrp-linked-antibody/7076
https://www.cellsignal.com/products/secondary-antibodies/anti-mouse-igg-hrp-linked-antibody/7077
https://www.thermofisher.com/antibody/product/Chicken-anti-Rabbit-IgG-H-L-Cross-Adsorbed-Secondary-Antibody-Polyclonal/A-21441
https://www.thermofisher.com/antibody/product/Goat-anti-Mouse-IgG-H-L-Highly-Cross-Adsorbed-Secondary-Antibody-Polyclonal/A32723
https://www.thermofisher.com/antibody/product/Goat-anti-Mouse-IgG-H-L-Highly-Cross-Adsorbed-Secondary-Antibody-Polyclonal/A11032
https://www.thermofisher.com/antibody/product/Goat-anti-Mouse-IgG-H-L-Highly-Cross-Adsorbed-Secondary-Antibody-Polyclonal/A11006

Eukaryotic cell lines

Policy information about cell lines and Sex and Gender in Research

Cell line source(s)	All NSCLC cell lines H1299, A549, Calu-6, SK-MES-1, H2006, H460 and H1437 were purchased from ATCC.
Authentication	Cell lines were authenticated by STR profiling in Genomics shared resources at OSUCCC
Mycoplasma contamination	The cell lines were tested negative for Mycoplasma contamination
Commonly misidentified lines (See ICLAC register)	No misidentified cell lines were used in this study.

Animals and other research organisms

Policy information about studies involving animals; ARRIVE guidelines recommended for reporting animal research, and Sex and Gender in Research

Laboratory animals	4-6 weeks old Athymic male and female mice, NOD scid gamma mice.
Wild animals	No wild animals were used in this study.
Reporting on sex	Both male and female mice were used.
Field-collected samples	No field collected samples were used in this study.
Ethics oversight	Institutional Animal Care and Use Committee of The Ohio State University (Columbus, OH).

Note that full information on the approval of the study protocol must also be provided in the manuscript.

Plants

Seed stocks	*Report on the source of all seed stocks or other plant material used. If applicable, state the seed stock centre and catalogue number. If plant specimens were collected from the field, describe the collection location, date and sampling procedures.*
Novel plant genotypes	*Describe the methods by which all novel plant genotypes were produced. This includes those generated by transgenic approaches, gene editing, chemical/radiation-based mutagenesis and hybridization. For transgenic lines, describe the transformation method, the number of independent lines analyzed and the generation upon which experiments were performed. For gene-edited lines, describe the editor used, the endogenous sequence targeted for editing, the targeting guide RNA sequence (if applicable) and how the editor was applied.*
Authentication	*Describe any authentication procedures for each seed stock used or novel genotype generated. Describe any experiments used to assess the effect of a mutation and, where applicable, how potential secondary effects (e.g. second site T-DNA insertions, mosiacism, off-target gene editing) were examined.*

Flow Cytometry

Plots

Confirm that:

- [x] The axis labels state the marker and fluorochrome used (e.g. CD4-FITC).
- [x] The axis scales are clearly visible. Include numbers along axes only for bottom left plot of group (a 'group' is an analysis of identical markers).
- [x] All plots are contour plots with outliers or pseudocolor plots.
- [x] A numerical value for number of cells or percentage (with statistics) is provided.

Methodology

Sample preparation	Cell cycle analysis. Cells were grown for 2-3 generations in multiwall plates and logarithmically growing cells were harvested by trypsinization and fixed in 70% methanol at -20°C overnight. After washing in 1× PSB twice cells were resuspended in 1× PSB containing 50 µg/ml PI & 100 µg/ml RNase A and incubated at 37°C for 1hr before analyzing on an BD Fortessa instrument using FACSDIVA™ software (BD Biosciences).<br>BrdU incorporation assay. BrdU incorporation assay was performed using PhaseFlow™ BrdU kit from Bio Legend (cat. 370704) according to manufacturer's protocol.<br>BrdU incorporation assay. BrdU incorporation assay was performed using PhaseFlow™ BrdU kit from Bio Legend (cat. 370704) according to manufacturer's protocol.<br>BrdU incorporation assay. BrdU incorporation assay was performed using PhaseFlow™ BrdU kit from Bio Legend (cat. 370704) according to manufacturer's protocol.<br>Organoid culture. Isolated tumor cells were then subjected to depletion of the mouse cell contamination using a Mouse cell depletion kit (Miltenyi Biotech; Cat# 130-104-694). The purity of the human cancer cells was verified using Human-EpCAM (CD326) (130-111-000) from Miltenyi Biotech.<br>Annexin V/PI apoptosis assay. Apoptosis in cultured cells was measured using an eBioscience™ Annexin V Apoptosis Detection Kit (Invitrogen; #Cat- 88-8102-72) according to the manufacturer's protocol.
Instrument	BD LSR Fortessa
Software	BD FACS DIVA; FlowJo v.10.6.1
Cell population abundance	Single cell abundance was accessed through FSC and SSC axis.

Gating strategy	For validation of tumor cell isolation for PDX model; PI negative Epcam Positive cells were gated.

☒ Tick this box to confirm that a figure exemplifying the gating strategy is provided in the Supplementary Information.
